# Candidate Chinese Herbal Medicine Alleviates Methamphetamine Addiction *via* Regulating Dopaminergic and Serotonergic Pathways

**DOI:** 10.3389/fnmol.2022.874080

**Published:** 2022-03-29

**Authors:** Qin Ru, Qi Xiong, Xiang Tian, Congyue Xu, Can Li, Lin Chen, Yuxiang Wu

**Affiliations:** ^1^Wuhan Institutes of Biomedical Sciences, School of Medicine, Jianghan University, Wuhan, China; ^2^Department of Health and Physical Education, Jianghan University, Wuhan, China

**Keywords:** Chinese herbal medicine, methamphetamine, addiction, network pharmacology, dopamine, serotonin

## Abstract

Methamphetamine (METH) addiction and its induced mental disorders have become a severe worldwide problem. A candidate Chinese herbal medicine (CCHM) in our lab had therapeutic effects on METH-induced locomotor sensitization, however, its chemical and pharmacological profiles remain to be elucidated. The current study aimed to investigate the effect of CCHM on conditioned place preference (CPP) induced by METH and screen the main active ingredients and key targets by using network pharmacology and molecular docking methods. Kyoto Encyclopedia of Genes and Genomes (KEGG) enrichment, Gene ontology (GO) analysis and protein-protein interaction (PPI) network were performed to discover the potential mechanisms. Results showed that CCHM could significantly inhibit METH-induced CPP behaviors in mice. A total of 123 components and 43 targets were screened. According to the network pharmacology analysis, ten hub targets including D(2) dopamine receptor (DRD2) and 5-hydroxytryptamine receptor 3A (HTR3A) were screened. GO analysis and KEGG enrichment indicated that mechanisms of CCHM treatment of METH addiction were related to multiple pathways such as dopaminergic synapse and serotoninergic synapse. Western blot results showed that the protein expressions of DRD2 in nucleus accumbens and prefrontal cortex were significantly decreased in METH group, while the protein expressions of HTR3A were significantly increased. These changes caused by METH could be prevented by CCHM pretreatment. The results of molecular docking displayed that the five active ingredients such as (S)-Scoulerine, Hyndarin, and Beta-Sitosterol had good affinities with DRD2 and HTR3A. In conclusion, this study constructed the CCHM’s pharmacologic network for treating METH addiction based on the method of network analysis and experimental verification, and analyzed its major active ingredients and potential targets, indicating a new direction for further revealing its mechanisms of effect on METH addiction.

## Graphical Abstract

Conditioned place preference (CPP) test was used to assess the potential therapeutic effects of CCHM on METH addiction. The active components and hub targets, which were screened by the network pharmacology analysis, were verified by further experiments.

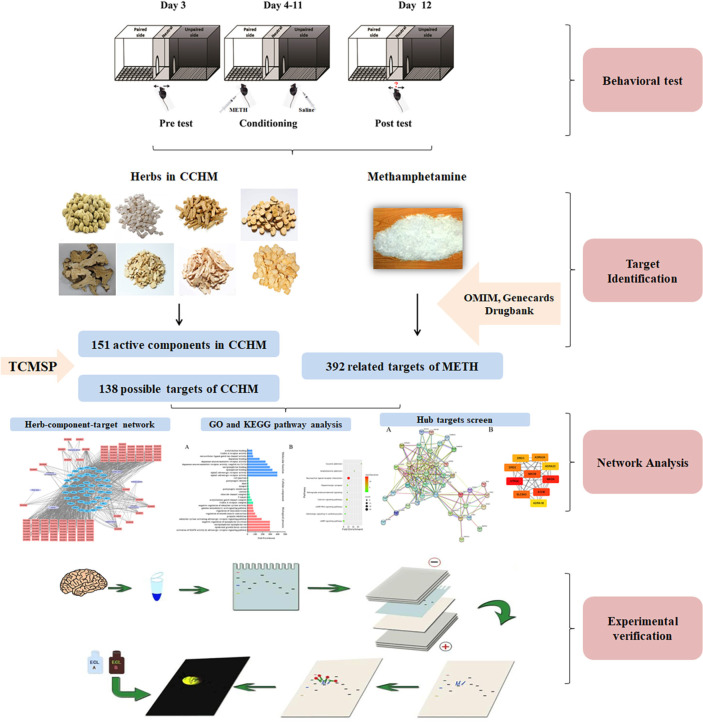

## Introduction

Drug addiction is a severe worldwide social problem, which has attracted great attention. Compared with drugs extracted from plants such as heroin and cocaine, the proportion of people who abuse amphetamine-type synthetic drugs is increasing year by year. According to reports released by United Nations Office on Drugs and Crime and the Ministry of Public Security of China, methamphetamine (METH) has become the most commonly abused amphetamine-type stimulant in Southeast Asia and North America. METH can easily enter the central nervous system through the blood-brain barrier, so its effect on brain is more evident than its peripheral effect (Paulus and Stewart, 2020). Studies have shown that METH abuse could seriously damage the structure and function of neurons and gliocyte in the prefrontal cortex, nucleus accumbens, hippocampus, and other brain regions of addicts, and cause a variety of central nervous system diseases, such as cognitive impairment, depression, and mental disorders (Zhang et al., 2018; Nie et al., 2020). The brain structure and function of most addicts cannot recover even after a prolonged period of abstinence, and the persistent mental disorders and negative emotions could also trigger the continuous drug-seeking behaviors, which led to relapse (Glasner-Edwards et al., 2009). METH addiction not only seriously affected the physical and mental health of abusers, but also led to crime, which has become a social problem endangering public security. Although antipsychotic drugs have been used to control the psychiatric symptoms caused by addiction, there is still a lack of drugs with definite efficacy on METH addiction in clinic. Therefore, the research and development of drugs for the prevention and treatment of METH addiction with better efficacy and fewer side effects has become the focus in drug abuse field.

The mechanism of METH addiction remains unclear, and multiple brain regions such as the prefrontal cortex and nucleus accumbens are involved, therefore, traditional Chinese medicine has become the focus of development of anti-addiction drugs due to its multi-system and multi-target mechanism (Chen et al., 2021). Our previous work have shown that a candidate Chinese herbal medicine (CCHM), which was composed of 8 traditional Chinese medicines including *Corydalis yanhusuo* and Codonopsis, could significantly improve the learning and memory impairment induced by METH, and inhibit the acquisition and expression of METH-induced locomotor sensitization (Can et al., 2019; Jiaming et al., 2019). The results of acute and long-term toxicity tests showed that CCHM had no detectable toxic effects (Can, 2019). However, as a Chinese herbal compound, the composition of CCHM is complex. A large number of experiments are still needed to analyze its main active ingredients and possible mechanisms. Based on the theory of systems biology, network pharmacology has the characteristics of systematic and holistic, and it is similar to the mechanism of traditional Chinese medicine in treating diseases. Network pharmacology could reveal the complex biological network relationships between drugs, targets, and diseases, analyze and predict the pharmacological mechanism of candidate drugs (Guo et al., 2019). Therefore, this study aimed to analyze the main active ingredients and critical targets of CCHM in the treatment of METH addiction based on the network pharmacology and molecular docking, and to provide the evidence for the clinical application of CCHM.

## Materials and Methods

### Animals

**FIGURE 1:**
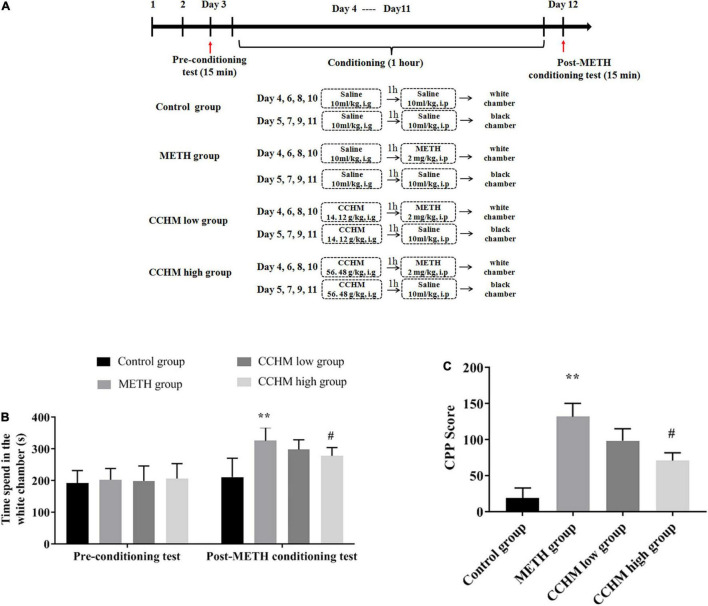
Effect of candidate Chinese herbal medicine (CCHM) on Methamphetamine (METH)-induced conditioned place preference (CPP) in mice. **(A)** The experiment procedure of CPP test. Mice received METH (2 mg/kg, i.p) or saline (10 ml/kg, i.p) injections following 1 h pretreatments of CCHM (14.12, 56.48 g/kg, i.g) or saline (10 ml/kg, i.g) treatments. **(B)** Time spent in the white chamber was collected before and after METH abuse. **(C)** CPP score was calculated by subtracting the time spent in the drug-paired compartment in the pre-conditioning test from that after METH exposure. Data were presented as mean ± SE, **p < 0.01, compared with control group; ^#^*p* < 0.05, compared with METH group, *n* = 10.

Male C57BL/6 mice (2 months old, weighing 18∼22 g) were purchased from Beijing Charles River Laboratories (Beijing, China). Mice were housed in a temperature-controlled room with a humidity of 45∼55% and subjected to a 12-h cycle of alternating light and dark. All mice had free access to food and water, and were pre-adapted to the environment for at least 3 days before the experiment. All experiment procedures were approved by the Ethics Committee of Jianghan University. As shown in Figure 1A, the mice were randomly divided into four groups: the control group, the METH group, CCHM low group, and CCHM high group. METH was offered by Hubei Public Security Bureau and was dissolved in saline, and CCHM was decocted and condensed into extract before use. Mice were intraperitoneally injected with METH (2 mg/kg) or saline following 1 h intragastric administration of CCHM (14.12, 56.48 g/kg) or saline. The dosage of CCHM was selected based on our published articles (Can et al., 2019).

### The Conditioned Place Preference Apparatus and Procedure

The conditioned place preference (CPP) facility was brought from Shanghai Xinruan Information Technology Co., Ltd. (Shanghai, China) and comprised three compartments. One chamber had a grid floor and black walls (L × W × H: 35 cm × 35 cm × 35 cm). Another same-sized chamber had a mesh floor and white walls. The middle chamber had a flat, gray floor and gray walls (L × W × H: 15 cm × 35 cm × 35 cm) and is connected to the other two compartments by removable doors. The white and black chamber had a ceiling light and camera, respectively. The general activity in the facility was monitored by video, and the time spent in each chamber was recorded using XR-XT401 software.

The CPP procedure used in this experiment was performed as previously described (Yang G. et al., 2020). The pretreatment period lasted from day 1 to day 3. Mice were placed in the middle chamber and then moved freely between the black and white chambers for 15 min per day. The time spent in each chamber on the third day was used as pre-processing data. The most visited chamber (black chamber) was designated as the preferred compartment and the other compartment (white chamber) was defined as drug compartment. Each animal received individual CPP training. The CPP training period lasted for 8 days (day 4∼day 11) and included four METH sessions and four saline sessions. The saline- and drug-paired compartments were separated by closed doors. On day 4, 6, 8, and 10, mice in METH or CCHM group were pretreated with saline or CCHM for 1 h and then confined to a drug-paired chamber (white chamber) for 1 h after intraperitoneally injected with 2 mg/kg METH. On days 5, 7, 9, and 11, the mice in METH or CCHM group were given saline or CCHM respectively and confined to the saline paired chamber (black chamber) for 1 h after saline injection. After each session, the floor and walls of each chamber were wiped with 75% ethanol. On day 12, the post-adaptation test was conducted for 15 min, and each mouse was placed in the middle chamber and then moved freely between the black and white chambers. Preference (CPP score) was calculated by subtracting the time spent in the white chamber on day 3 from the time spent in the white chamber on day 12 (Yang C. et al., 2020). CPP score reflects the difference in the time spent in the post-conditioning and pre-conditioning phases of METH.

### Identification of Candidate Components in Candidate Chinese Herbal Medicine

Candidate Chinese herbal medicine was composed of eight Chinese medicinal herbs, including *C. yanhusuo*, *Poria cocos*, *Codonopsis pilosula*, Licorice, *Atractylodes macrocephala* Koidz, *Astragalus membranaceus*, *Angelica sinensis*, and American ginseng. The components of CCHM were retrieved from Traditional Chinese medicine system pharmacology (TCMSP) database.^1^ Oral Chinese medicine must overcome the obstacles of absorption, distribution, metabolism, and excretion (ADME) process in order to be effective, so oral bioavailability (OB) is one of the most important pharmacokinetic parameters in ADME. High OB is usually an important index for determining drug similarity (DL) of active substances (Guo et al., 2019). Substances with OB no less than 30% are considered to have a higher OB. DL index, as a qualitative concept used to estimate the medicinal properties of molecules in drug design, can be used for rapid screening of active substances (Tao et al., 2013; He et al., 2019). In the Drugbank database, the average DL index is 0.18. Substances with a DL index no less than 0.18 have higher medicinal properties. Therefore, compounds with OB ≥ 30% and DL index ≥ 0.18 and blood brain barrier (BBB) ≥ −0.3 in CCHM were selected as active substances in this study. The possible targets of the active substances of CCHM were searched from TCMSP database. The Uniprot database^2^ and the bioDBnet website^3^ were used to verify the gene symbols corresponding to the possible targets.

### Screening of Possible Targets of Methamphetamine Addiction

Potential targets involved in METH addiction were collected from OMIM,^4^ Drugbank,^5^ and Genecards^6^ databases. Potential targets from different databases were combined and the duplicates has been deleted.

### Network Analysis of Active Compounds and Potential Targets

The potential targets of active compounds in CCHM were mapped with METH addiction-related targets on the Bioinformatics and Evolutionary Genomics website.^7^ Cytoscape 3.8.0. software was used to construct the “herb-compound-target” network (Han et al., 2020). In these graphical networks, the compounds and proteins were expressed as nodes, whereas the compound-target interactions were expressed as edges.

### Network Analysis of Protein-Protein Interaction

Potential targets of CCHM on METH addiction were analyzed by online STRING 11.0^8^ to construct protein-protein interaction (PPI) network. The network was visualized with Cytoscape (3.8.0) and CytoHubba, a plug-in in Cytoscape, to filter the modules from the PPI network and to obtain the most important hub genes based on the degree score.

### Gene Ontology and Kyoto Encyclopedia of Genes and Genomes Enrichment of Hub Genes

To further characterize the molecular mechanism of CCHM on METH addiction, the DAVID website^9^ was used to conduct the GO analysis and KEGG enrichment of hub targets. Top terms of GO analysis and KEGG enrichment with thresholds of *p*-values ≤ 0.05 were chosen in functional annotation clustering.

### Literature Verification

Literature retrieval was used to excavate and analyze the active components and possible mechanisms of CCHM in the treatment of METH addiction. This may provide literature verification for prediction results of the network pharmacology.

### Western Blotting Analysis

Mice were sacrificed by cervical dislocation under anesthesia after behavioral testing. Protein extracts in nucleus accumbens and prefrontal cortex were isolated, lysed, and the supernatant was collected after centrifugation. BCA Protein Assay Kit was used to detect the concentration of protein extracts. Protein samples were separated and electroblotted onto the PVDF membrane. After blocking, membranes were incubated with primary antibodies followed by a horseradish peroxide conjugated secondary antibody. Enhanced chemiluminescence was used to visualize the band, and the intensity of each sample was determined quantitatively using Image J. Actin was used as loading control, and the results were normalized to the control group.

### Molecule Docking of Main Active Compounds of Candidate Chinese Herbal Medicine With Hub Targets

The amino acid sequence of the two hub targets D(2) dopamine receptor (DRD2) and 5-hydroxytryptamine receptor 3A (HTR3A) were downloaded from the PDB protein structure database.^10^ Furthermore, AutoDock v.4.2.6 was used to add the hydrogens, calculate charges, and merge the non-polar hydrogens of protein structure (Zulkipli et al., 2020). Next, the protein file was saved in PDBQT format. The three-dimensional (3D) structures of main active compounds were downloaded from the PubChem structure database.^11^ The SDF format of main active compounds was converted to PDB format using OpenBabel 2.4.1 (Kumar et al., 2020). AutoDock v.4.2.6 was used to convert the PDB format of resveratrol to PDBQT format and for molecular docking of main active compounds with hub targets. PyMol software was used to conduct a visual analysis of docking results.

### Statistical Analysis

Statistical analysis was processed with SPSS 23.0 and Prism 8 software. Data were shown as the mean ± SE and analyzed using one-way ANOVA and LSD *post-hoc* test. Differences between groups were considered to be statistically significant if values of *p* < 0.05.

## Results

### Candidate Chinese Herbal Medicine Inhibited Methamphetamine-Induced Conditioned Place Preference Behavior in Mice

Conditioned place preference, a classic model widely used to assess drug-seeking behaviors, was used in this study to assess the preference for visual and tactile cues associated with METH after being administered with varying doses of CCHM. As shown in Figure 1B, mice in the control group did not produce motivational effects before or after place conditioning, which set the baseline measurement for the CPP test. METH administration at 2 mg/kg significantly increased preference of white chamber compared to the controls (*p* < 0.01). As shown in Figure 1C, compared to the METH group, pretreatment of CCHM reduced METH-induced motivational effects (*p* < 0.05), and CPP scores decreased with the increase of CCHM dose. Taken together, the results suggested that 2 mg/kg of METH was sufficient to induce CPP behaviors, and the co-administration of CCHM attenuated the acquisition of METH-induced CPP in mice.

### Identification of Bioactive Compounds and Possible Targets in Candidate Chinese Herbal Medicine

A total of 163 compounds in CCHM obtained from TCMSP database met the requirement of OB ≥ 30%, DL index ≥ 0.18 and BBB ≥ −0.3, and 48 of which belong to *C. yanhusuo*, six to *P. cocos*, 16 to *C. pilosula*, 69 to Licorice, four to *A. macrocephala* Koidz, 12 to *A. membranaceus*, two to *A. sinensis*, and six to American ginseng. After eliminating the overlaps, 151 compounds were chosen as candidate bioactive compounds for further analyses, and the detailed information was shown in Table 1 and Supplementary Material 1. Among the 151 candidate bioactive components, 2,492 possible protein targets were retrieved from the TCMSP database. The detailed information was shown in Supplementary Material 2. After eliminating the overlaps, 138 protein targets were obtained for further analyses.

**TABLE 1:** Information of “Herb-active component-predictive target” for candidate Chinese herbal medicine (CCHM).

Herb	Number of active components	Number of predictive target
*Corydalis yanhusuo*	48	942
*Poria cocos*	6	27
*Codonopsis pilosula*	16	139
Licorice	69	1276
*Atractylodes macrocephala* Koidz	4	22
*Astragalus membranaceus*	12	102
*Angelica sinensis*	2	69
American ginseng	6	65

### Targets Identification of Candidate Chinese Herbal Medicine on Methamphetamine Addiction

Sixty METH addiction-related targets were collected from OMIM, 356 targets were collected from the Genecards database, and 11 targets were obtained from Drugbank database. After eliminating the overlaps, 392 targets were obtained for further analyses. The detailed information was shown in Supplementary Material 3. Then, these protein targets of CCHM were mapped with METH addiction-related targets using the Bioinformatics and Evolutionary Genomics website. As a result, 43 proteins were selected, corresponding to 123 active ingredients of CCHM and the detailed information of 43 proteins was shown in Supplementary Material 4.

### Construction of “Herb-Compound-Target” Network

**FIGURE 2:**
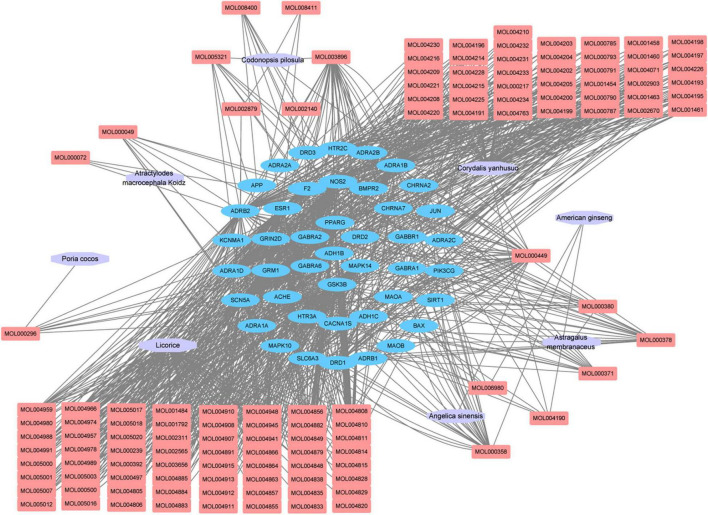
“Herb-compound-target” network of candidate Chinese herbal medicine (CCHM) in the treatment of methamphetamine (METH) addiction. The purple polygons represented different herbs. The pink squares represented active compounds of herbs, and the blue circle represented potential protein targets. The edges represent the interactions between them.

Cytoscape software was used to construct the of “herb-compound-target” network, as shown in Figure 2. Among these bioactive compounds, the top 10 compounds were 7-*O*-Methylisomucronulatol, 7-Methoxy-2-Methylisoflavone, Formononetin, Beta- Sitosterol, *S*-Scoulerine, Isocorypalmine, Leonticine, Medicarpin, Licochalcone, and Hyndarin. The detailed information was shown in Table 2. These bioactive components may be the main active compounds of CCHM in treating METH addiction.

**TABLE 2:** Top ten components of candidate Chinese herbal medicine (CCHM) on methamphetamine (METH) addiction.

Molecule name	Number in TCMSP	Number of possible targets in TCMSP	Number of targets mapped with METH addiction	Herb
7-*O*-Methylisomucronulatol	MOL000378	45	17	*Astragalus membranaceus*
7-Methoxy-2-Methylisoflavone	MOL003896	43	17	*Codonopsis pilosula*/Licorice
Formononetin	MOL000392	39	13	Licorice
Beta-Sitosterol	MOL000358	38	12	American ginseng /*Angelica sinensis*
(S)-Scoulerine	MOL000217	36	12	*Corydalis yanhusuo*
Isocorypalmine	MOL000790	36	13	*Corydalis yanhusuo*
Leonticine	MOL004215	35	14	*Corydalis yanhusuo*
Medicarpin	MOL002565	34	12	Licorice
Licochalcone A	MOL000497	32	9	Licorice
Hyndarin	MOL004071	32	12	*Corydalis yanhusuo*

### Gene Ontology and Pathway Enrichment Analysis

**FIGURE 3:**
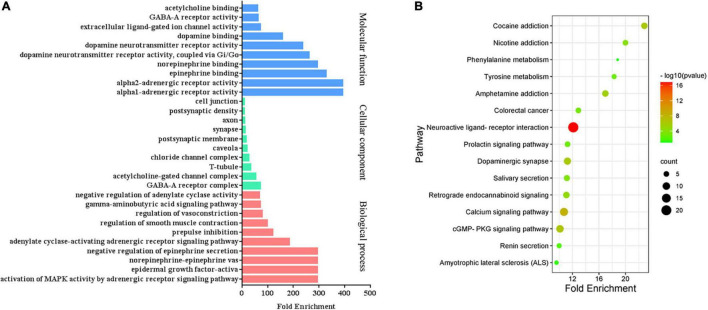
Clustering analysis of possible targets of candidate Chinese herbal medicine (CCHM) in the treatment of methamphetamine (METH) addiction. **(A)** Gene ontology (GO) analysis of the possible targets. **(B)** Kyoto encyclopedia of genes and genomes (KEGG) enrichment analysis of the possible targets. Counts referred to the number of the enriched targets.

To identify the biological characteristics of putative targets of CCHM on METH addiction in detail, the GO and KEGG enrichment of involved targets were conducted *via* the functional annotation tool of DAVID Bioinformatics Resources 6.8. There were 116 terms of biological process (BP), 20 terms of cellular component (CC), and 31 terms of molecular function (MF) in total, which met the requirements of Count ≥ 2 and *p*-value ≤ 0.05. The detailed information was shown in Supplementary Material 5. The top 10 significantly enriched terms in BP, CC, and MF categories were shown in Figure 3A, which indicated that CCHM may regulate neuron functions *via* adrenergic receptor signaling pathway, dopamine neurotransmitter receptor activity, dopamine binding to exert its therapeutic effects on METH addiction. To explore the underlying involved pathways of CCHM on METH addiction, KEGG pathway analysis of involved targets was conducted. The detailed pathway information of CCHM on METH addiction was shown in Supplementary Material 6. The top 15 significantly enriched pathways were shown in Figure 3B. The neuroactive ligand-receptor interaction pathways exhibited the largest number of involved targets (21 counts).

### Screen of Hub Genes

**FIGURE 4:**
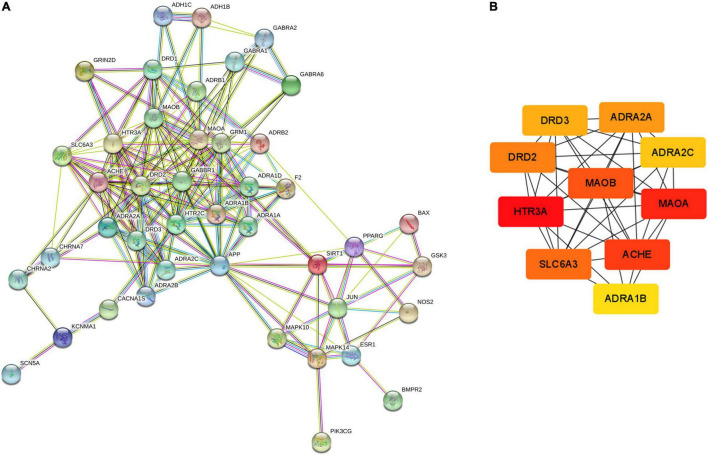
Protein-protein interaction (PPI) network and Hub gene screening map of targets. **(A)** PPI network exported from STRING database. **(B)** Key subnetwork of top 10 nodes analysed by CytoHubba.

The PPI network of possible targets involved in the treatment of CCHM on METH addiction was constructed using STRING (Figure 4A). There were 43 nodes and 200 edges, and the average node degree was 9.3. The average local clustering coefficient was 0.637, and the *p*-value of PPI enrichment was less than 1.0e-16. To obtain the hub genes in the PPI network, these node pairs were entered into the Cytoscape software. The scores of nodes were calculated by cytoHubba, and the top 10 hub genes were shown in Figure 4B.

**FIGURE 5:**
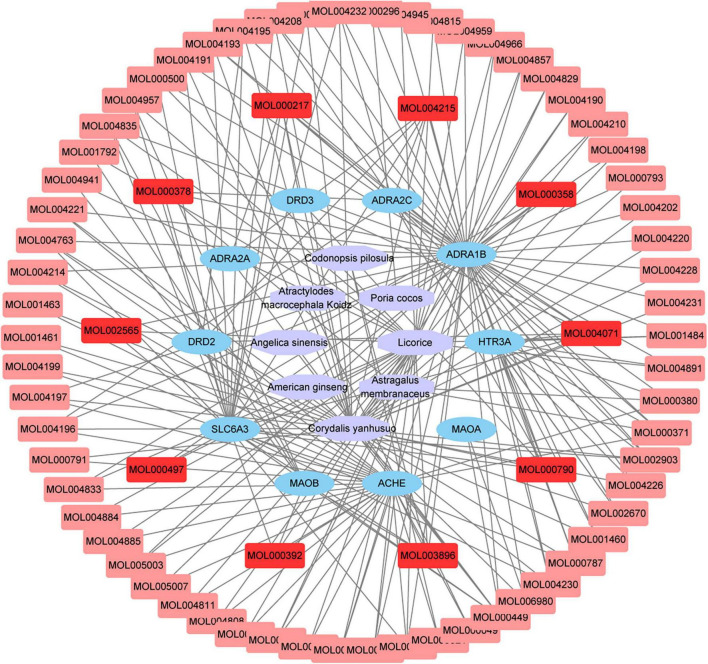
Network chart of “Herb-Main active compounds-hub targets.” The purple polygons represented different herbs. The blue circle represented potential protein targets. The pink squares represented active compounds of herbs, and the red squares represented main active compounds. The edges represent the interactions between them.

The hub gene symbols, full names, and functions were shown in Table 3. These 10 key targets corresponded to 75 major active components of CCHM. The network diagram of “herb-major active component-hub target” constructed by Cytoscape software was shown in Figure 5.

**TABLE 3:** Detail information of ten hub genes of candidate Chinese herbal medicine (CCHM) on methamphetamine (METH) addiction.

Gene symbol	Full names	Functions
HTR3A	5-hydroxytryptamine receptor 3A	It is one of receptors for 5-hydroxytryptamine (serotonin), which when activated causes fast, depolarizing responses in neurons.
MAOA	Monoamine oxidase A	It catalyzes the oxidative deamination of biogenic and xenobiotic amines and has important functions in the metabolism of neuroactive and vasoactive amines. MAOA preferentially oxidizes biogenic amines such as 5-hydroxytryptamine (5-HT), norepinephrine and epinephrine. MAOB preferentially degrades benzylamine and phenylethylamine.
MAOB	Monoamine oxidase B	
ACHE	Acetylcholinesterase	It terminates signal transduction at the neuromuscular junction by rapid hydrolysis of the acetylcholine released into the synaptic cleft.
DRD2	D(2) dopamine receptor	Dopamine receptor whose activity is mediated by G proteins which inhibit adenylyl cyclase.
ADRA2A	Alpha-2A adrenergic receptor	It mediates the catecholamine-induced inhibition of adenylate cyclase through the action of G proteins.
SLC6A3	Sodium-dependent dopamine transport	It terminates the action of dopamine by its high affinity sodium-dependent reuptake into presynaptic terminals.
DRD3	D(3) dopamine receptor	Dopamine receptor whose activity is mediated by G proteins which inhibit adenylyl cyclase.
ADRA2C	Alpha-2C adrenergic receptor	It mediates the catecholamine-induced inhibition of adenylate cyclase through the action of G proteins.
ADRA1B	Alpha-1B adrenergic receptor	It mediates its action by association with G proteins that activate a phosphatidylinositol-calcium second messenger system.

### Gene Ontology and Pathway Enrichment Analysis of Hub Genes

**FIGURE 6:**
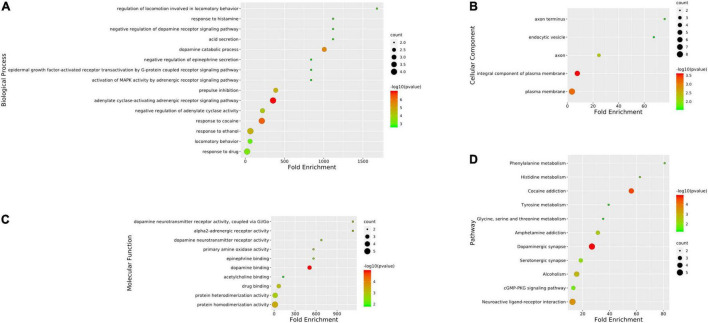
Gene ontology (GO) analysis and kyoto encyclopedia of genes and genomes (KEGG) enrichment of hub targets of candidate Chinese herbal medicine (CCHM) in the treatment of methamphetamine (METH) addiction. **(A)** The top 15 significantly enriched terms in BP enrichment analysis of the hub genes. **(B)** The top five significantly enriched terms in CC enrichment analysis of the hub genes. **(C)** The top 10 significantly enriched terms in MF enrichment analysis of the hub genes. **(D)** The top 11 significantly enriched terms in KEGG enrichment analysis. Counts refer to the number of the enriched genes.

The GO analyses and KEGG enrichment of hub genes were conducted *via* DAVID 6.8. There were respectively 51 BP, 5 CC, and 10 MF terms in total, which met the requirements of Count ≥ 2 and *p*-value ≤ 0.05. The detailed information was shown in Supplementary Material 7. The top enriched terms in BP, CC, and MF categories were shown in Figure 6. Among them, the results of BP (Figure 6A) showed that the treatment of METH addiction by CCHM was mainly related to adenylate cyclase-activating adrenergic receptor signaling pathway, response to cocaine and dopamine catabolic process. The results of CC (Figure 6B) showed that its treatment was mainly related to plasma membrane, axon, axon terminus, and so on. The results of MF (Figure 6C) showed that items including dopamine binding, protein homodimerization activity, and dopamine neurotransmitter receptor activity had close relationship with the treatment of METH addiction by CCHM, and the genes involved were shown in Table 4. There were 11 KEGG pathways met the requirements of Count ≥ 2 and *p*-values ≤ 0.05. The detailed pathway information of hub genes was shown in Supplementary Material 8 and Figure 6D, which indicated that CCHM may regulate METH addiction *via* dopaminergic synapse, cocaine addiction, neuroactive ligand-receptor interaction, amphetamine addiction, and serotonergic synapse.

**TABLE 4:** Results of kyoto encyclopedia of genes and genomes (KEGG) analysis on hub targets of candidate Chinese herbal medicine (CCHM) in the treatment of methamphetamine (METH) addiction.

Pathway	Counts	Genes
Dopaminergic synapse	5	DRD3, DRD2, SLC6A3, MAOA, MAOB
Cocaine addiction	4	DRD2, SLC6A3, MAOA, MAOB
Neuroactive ligand-receptor interaction	5	DRD3, DRD2, ADRA2A, ADRA1B, ADRA2C
Alcoholism	4	DRD2, SLC6A3, MAOA, MAOB
Amphetamine addiction	3	SLC6A3, MAOA, MAOB
Serotonergic synapse	3	MAOA, MAOB, HTR3A
cGMP-PKG signaling pathway	3	ADRA2A, ADRA1B, ADRA2C

### Literature Verification

It was found that Scoulerine could significantly inhibit the CPP behavior and alleviate the anxiety-like behavior caused by METH (Mi et al., 2016). Hyndarin, also known as *L*-tetrahydropalmatine, had significant inhibitory effects on locomotor sensitization, CPP behavior, self-administration behavior, and relapse behavior induced by METH (Zhao et al., 2014; Gong et al., 2016; Su et al., 2020), and hyndarin could also improve METH-induced learning and memory impairment (Cao et al., 2018). Studies have shown that hyndarin could improve METH-induced locomotor sensitization by regulating the activity of 5-HT neurons and the expression of dopamine D3 receptor (Yun, 2014b). These literature reports further validated the network pharmacological prediction results. The relationship between the other eight compounds and METH addiction has not been reported.

### Protein Expression Validation of Predicted Target Genes

**FIGURE 7:**
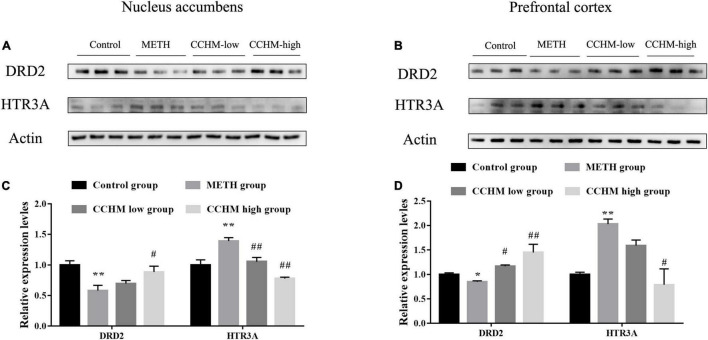
Candidate Chinese herbal medicine (CCHM) administration inhibited methamphetamine (METH)-induced expression changes of D(2) dopamine receptor (DRD2) and 5-hydroxytryptamine receptor 3A (HTR3A) in the nucleus accumbens and prefrontal cortex. **(A)** Representative bands of DRD2 and HTR3A in the nucleus accumbens. **(B)** Representative bands of DRD2 and HTR3A in the prefrontal cortex. **(C)** METH significantly decreased expression of DRD2 and increased expression of HTR3A, while CCHM pretreatment could greatly upregulate DRD2 expression and downregulate HTR3A expression. **(D)** The expression of DRD2 notably decreased and the level of HTR3A increased in METH group, which could be reverse by CCHM pretreatment. *n* = 3 for per group, *p < 0.05, **p < 0.01 compared to the control group, #*p* < 0.05, ##*p* < 0.01 compared to the METH group.

To further verify the effects of CCHM on the expressions of the potential targets identified *via* network pharmacology, the expression of DRD2 and HTR3A in nucleus accumbens and prefrontal cortex, which play important roles in the reward system, were examined using Western blotting. As shown in Figures 7A,C, compared with the control group, the protein level of DRD2 in nucleus accumbens was significantly decreased in the METH group, while the protein expression of HTR3A was greatly increased. Furthermore, pretreatment with CCHM could prevent METH-induced changes of DRD2 and HTR3A protein expressions in nucleus accumbens. Similar results were also found in the prefrontal cortex (Figures 7B,D). These results validated that CCHM may inhibit the METH induced abuse mainly through dopaminergic synapse and serotonergic synapse pathways.

### Molecular Docking Main Active Compounds and Hub Targets

**FIGURE 8:**
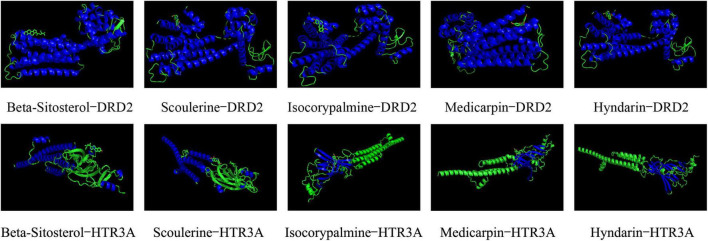
Docking conformations of main active components with D(2) dopamine receptor (DRD2) and 5-hydroxytryptamine receptor 3A (HTR3A).

To screen the main active compounds in CCHM, the docking results of 10 major active components of CCHM acting on DRD2 and HTR3A were shown in Table 5 and Figure 8. Results of molecular docking showed that eight of the 10 major active components had good binding abilities with the potential targets of methamphetamine treatment (DRD2 and HTR3A), and β-Sitosterol, scoulerine, isocorypalmine, medicarpin, and hyndarin had better binding activities with the two target proteins, and the molecular conformational binding was stable, which may affect the downstream signaling pathway to participate in the treatment of METH addiction by binding with the two targets.

**TABLE 5:** Results of ligand-receptor protein molecular docking.

Molecule Name	Binding energy with DRD2/KJ mol^–1^	Binding energy with HTR3A/KJ mol^–1^
7-O-Methylisomucronulatol	–2.77	–2.92
Formononetin	–1.43	–3.52
Beta-Sitosterol	–2.94	–4.07
(S)-Scoulerine	–3.3	–3.96
Isocorypalmine	–3.42	–3.6
Leonticine	–1.87	–2.84
Medicarpin	–3.09	–4.28
Hyndarin	–3.54	–3.91

## Discussion

Methamphetamine is widely abused worldwide, and effective clinical treatment strategy for METH abuse is still urgently needed. CCHM is a traditional Chinese medicine developed for the treatment of METH addiction, and our previous results showed that CCHM could significantly inhibit the acquisition and expression of METH-induced behavioral sensitization (Can et al., 2019), indicating that CCHM had the potential to ameliorate METH addiction. In this study, we found that pre-treatments of CCHM could significantly inhibit METH-induced conditional place preference, which further verified that CCHM had the potential to be developed as a candidate drug for the treatment of METH addiction. However, as a multi-component Chinese herbal compound, the pharmacodynamics basis and mechanism of CCHM are still unclear. In this study, GO and KEGG analysis revealed that CCHM could regulate dopaminergic synapse, serotonergic synapse, and neuroactive ligand-receptor interaction pathways. Among 10 hub targets out of 43 genes by PPI analyses, we focused on two most significant genes DRD2 and HTR3A, and performed molecular docking to verify interactions between active compounds of CCHM and DRD2 (or HTR3A). Our results demonstrated the effectiveness of CCHM in the treatment of METH addiction from bioinformatics and experimental perspectives, and provided more evidence for its clinical application.

Long-term abuse of METH would damage the nervous system, resulting in rapid cognitive decline, anxiety, mental disorders, hyperalgesia, depression, etc. (Prakash et al., 2017). At the same time, long-term use of METH also inhibited immune function and increased susceptibility to infection (Harms et al., 2012; Peerzada et al., 2013). CCHM consists of eight Chinese medicinal herbs. *C. yanhusuo* had the effect of promoting blood circulation and relieving pain (Ingram, 2014; Zhou et al., 2019). *A. macrocephala* Koidz, *P. cocos*, American ginseng, Licorice, and *C. pilosula* could improve the body immunity and reduce the damage of METH to the central nervous system (Fu et al., 2016; Tian et al., 2019; Bai et al., 2020). *A. sinensis* and *A. membranaceus* had good sedation and tranquilizing effects (Chen et al., 2004; Park et al., 2009). Therefore, it is reasonable to speculate that different herbs in CCHM may play an effective synergistic role in regulating METH addiction and its various complications, and the cooperation among different components could be achieved from the distinct mode of actions to achieve a complementary pharmacological synergy.

In the current study, the components in CCHM with OB ≥ 30%, DL index ≥ 0.18 and BBB ≥ −0.3 were considered pharmacokinetically active as they are possibly absorbed and distributed in human brain. 123 components and 43 targets of CCHM for the treatment of METH addiction were screened by databases. Compounds with high-degree may account for the major therapeutic effects of CCHM on METH addiction. Ten major active components including Beta-Sitosterol, *S*-Scoulerine, Isocorypalmine, Leonticine, Hyndarin, and 10 hub targets including DRD2, HTR3A, and MAOA were screened out. Results of hub targets GO and KEGG enrichment analysis showed that dopaminergic synapses, cocaine addiction, neuroactive ligand-receptor interaction, alcoholism, and serotonergic synapse were the main possible pathways involved in the CCHM treatment of METH addiction.

The neural circuits that project from dopamine neurons in the ventral tegmental area to the nucleus accumbens and frontal cortex are called the mesolimbic dopamine system (MLDS). Numerous studies have identified the MLDS as a common reward circuit and main neuroanatomical basis for the rewarding or positive reinforcement of addictive drugs, involving in drug-induced reinforcement, pathological memory, craving, and relapse (Peters et al., 2021). Addictive substances affect the release of monoamine neurotransmitters such as dopamine, adrenaline and 5-hydroxytryptamine by acting on the reward system, and regulate the excitability of neurons. Under physiological conditions, the presynaptic release and reuptake of dopamine in dopaminergic neurons are in equilibrium (Volkow et al., 2017; Limanaqi et al., 2018). METH enters neurons through dopamine transporter, spurs presynaptic dopamine release and increases the concentration of dopamine in the synaptic cleft. Moreover, it could replace endogenous dopamine vesicles *via* combination of monoamine transporter in neurons, inhibit dopamine uptake, and promote the interaction of dopamine and receptors to produce neuroadaptive and addictive behaviors (Krasnova and Cadet, 2009; Shin et al., 2017). Michael et al. found that intravenous METH injection could significantly increase the levels of dopamine in the nucleus accumbens, and the locomotor activities of rats was consistent with the change of dopamine levels in the nucleus accumbens (Baumann et al., 2011), and reducing dopamine levels in the nucleus accumbens could reduce the locomotor activities of addicted mice (Yun, 2014a). These studies suggested that dopaminergic system played important roles in METH-induced addictive behaviors. Among the 10 hub targets screened in this study, SLC6A3 is a dopamine transporter, which regulates the reuptake of dopamine, and plays a role in maintaining the homeostasis of dopamine in the synaptic cleft. The expression of SLC6A3 in the striatum of METH abusers was significantly decreased, accompanied with reduced learning and memory ability and motor ability (Volkow et al., 2001b,c). After 4 weeks of drug withdrawal, the expression of dopamine transporter in the striatum of abusers gradually recovered, but it was still significantly lower than that of healthy subjects (Yuan et al., 2014). Dopamine transporter inhibitors such as JHW007 and tetrabenazine significantly reduced relapse and locomoter sensitization in METH-addicted rats (Meyer et al., 2011; Ferragud et al., 2014). DRD2 and DRD3 belong to dopamine receptors. DRD3 antagonists could significantly inhibit the reinforcement of METH and relapse (Chen et al., 2014; Sun et al., 2016). METH abuse has been associated with DRD2 and DRD3 deficits in the caudate nucleus and nucleus accumbens, and DRD2 receptor availability was lower in the METH abusers and may mediate impulsive temperament and thereby influence addiction (Volkow et al., 2001a; Lee et al., 2009; Groman et al., 2012; Wang et al., 2012; Okita et al., 2018). Animal experiment also confirmed that mRNA expression of DRD2 was significantly decreased after METH administration (Landa et al., 2012), suggesting poor DRD2 function may be a biomarker that predicts a greater likelihood for relapse. Therefore, we focused on the expression of DRD2 in mesolimbic dopamine system to further verify the results of bioinformatics analysis. Consistent with previous literatures, our results also proved that DRD2 in nucleus accumbens and prefrontal cortex was significantly decreased in METH group, while CCHM treatment could increase the levels of DRD2. Our findings combined with previous reports further verified the vital role of dopamine system in the prevention and treatment of METH addiction.

In addition to the dopamine system, 5-hydroxytryptamine, adrenaline system and their related metabolic enzymes are also the hub targets of CCHM in the prevention and treatment of METH addiction. MAOA and MAOB specifically oxidize and inactivate monoamine transmitters, and MAOA gene polymorphisms were associated with susceptibility to METH-induced mental disorders (Nakamura et al., 2009). ADRA2A, ADRA2C, and ADRA1B are adrenergic receptors. METH could increase the expression of ADRA2A in the hippocampus (Nishio et al., 2002), and downregulation of ADRA2A inhibited METH-induced hyperactivity (Nishio et al., 2003), and knockdown of ADRA1B had a protective effect on METH-induced neurotoxicity (Battaglia et al., 2003). It was reported that karbalatin, an inhibitor of the acetylcholinesterase AChE could also reduce METH self-administration behavior (De La Garza et al., 2008), suggesting that acetylcholine may be involved in METH addiction. HTR3A is one of the 5-hydroxytryptamine receptors. Electrophysiological results showed 0.1 μM METH increased 5-HT-induced inward peak current (*I*_5–HT_) in oocytes expressing HTR3A receptor, while high dose METH inhibited *I*_5–HT_, and HTR3A receptor antagonist MDL72222 had an inhibitory effect on the acquisition and expression of METH locomoter sensitization (Yoo et al., 2006). HTR3A receptor antagonist ondansetron had a protective effect on the neurotoxicity caused by METH (Lafuente et al., 2018). Among all the hub genes, HTR3A has the highest degree score in the cytoHubba analysis, therefore, the expression of HTR3A in mesolimbic dopamine system was also detected. Our results demonstrated that METH addiction significantly increased HTR3A expression in nucleus accumbens and prefrontal cortex, while CCHM treatment decreased the levels of HTR3A while inhibiting METH addiction, which were consistent with results in previous literatures. These findings further validated that CCHM may inhibit the METH induced abuse mainly through regulating serotonergic system.

In this study, 10 major active compounds were molecularly docked with DRD2 and HTR3A, two receptors with higher degrees in the hub targets. The results showed that most active compounds could effectively bind to DRD2 and HTR3A receptors. Among them, β-Sitosterol, Scoulerine, Isocorypalmine, Medicarpin, and Hyndarin had lower binding energy with DRD2 and HTR3A. These results were consistent with previous studies which showed Scoulerine and Hyndarin had the potential to inhibit METH addiction, and Isocorypalmine could reduce the behavioral sensitization and reward of cocaine in mice by acting on dopamine receptors (Xu et al., 2013). To the best of our knowledge, there are no related literature reports on β-Sitosterol and Medicarpin and drug addiction, and our results suggested they might be novel potential molecules for the treatment of drug addiction. Based on literature verification and results of molecular docking, β-Sitosterol, Scoulerine, Isocorypalmine, Medicarpin, and Hyndarin may be potential active components for the treatment of CCHM on METH addiction, and the underlying mechanism of action may be through DRD2 and HTR3A signaling pathways.

## Conclusion

To sum up, the pharmacological mechanism by which CCHM inhibited METH addiction was investigated based on the network pharmacology analysis, literature, and experimental validation. The results of the current study have shed light on further research on the pharmacodynamics and molecular mechanism of CCHM in the treatment of METH addiction and related mental disorders. In the future, *in vivo* and *in vitro* experiments are still needed to systematically verify the main active components and possible key targets in detail. The potential clinical therapeutic effects of CCHM may benefit from further studies.

## Data Availability Statement

The datasets presented in this study can be found in online repositories. The names of the repository/repositories and accession number(s) can be found in the article/Supplementary Material.

## Ethics Statement

The animal study was reviewed and approved by the Ethics Committee of Jianghan University.

## Author Contributions

QR, LC, and YW designed the study, analyzed the data, and prepared the manuscript together. QR, XT, CL, and QX performed the experiments. All authors have read and approved the final version of the manuscript.

## Conflict of Interest

The authors declare that the research was conducted in the absence of any commercial or financial relationships that could be construed as a potential conflict of interest.

## Publisher’s Note

All claims expressed in this article are solely those of the authors and do not necessarily represent those of their affiliated organizations, or those of the publisher, the editors and the reviewers. Any product that may be evaluated in this article, or claim that may be made by its manufacturer, is not guaranteed or endorsed by the publisher.

## Footnotes

1
http://tcmspw.com/tcmsp.php

2
http://www.Unitprot.org/

3
https://biodbnet-abcc.ncifcrf.gov/

4
https://www.omim.org/

5
https://www.drugbank.ca/

6
https://www.genecards.org/

7
http://bioinformatics.psb.ugent.be/webtools/Venn/

8
https://string-db.org/

9
https://david.ncifcrf.gov/

10
http://www.rcsb.org/

11
https://pubchem.ncbi.nlm.nih.gov/

## References

[B1] Bai R. B. Zhang Y. J. Fan J. M. Jia X. S. Li D. Wang Y. P. (2020). Immune-enhancement effects of oligosaccharides from *Codonopsis pilosula* on cyclophosphamide induced immunosuppression in mice. *Food Funct.* 11 3306–3315. 10.1039/c9fo02969a 32227014

[B2] Battaglia G. Fornai F. Busceti C. L. Lembo G. Nicoletti F. De Blasi A. (2003). Alpha-1B adrenergic receptor knockout mice are protected against methamphetamine toxicity. *J. Neurochem.* 86 413–421. 12871582 10.1046/j.1471-4159.2003.01867.x

[B3] Baumann M. H. Clark R. D. Woolverton W. L. Wee S. Blough B. E. Rothman R. B. (2011). *In vivo* effects of amphetamine analogs reveal evidence for serotonergic inhibition of mesolimbic dopamine transmission in the rat. *J. Pharmacol. Exp. Ther.* 337 218–225. 10.1124/jpet.110.176271 21228061 PMC3063744

[B4] Can L. (2019). *Analysis of Jieduyihao Fingerprint And Its Effect On Behavioral Sensitization Induced By Methamphetamine In Mice.* Wuhan: Jianghan University.

[B5] Can L. Lijun T. Yuanren S. Huaqiao X. Qin R. Mei Z. (2019). Effects of jieduyihao on methamphetamine-induced behavioral sensitization in mice. *J. Jianghan Univ. (Nat. Sci. Ed.)* 47 57–65.

[B6] Cao G. Zhang Y. Zhu L. Zhu J. Zhao N. Dong N. (2018). The inhibitory effect of levo-tetrahydropalmatine on the methamphetamine-induced spatial memory impairment in mice. *Neurosci. Lett.* 672 34–39. 29447954 10.1016/j.neulet.2018.02.018

[B7] Chen L. Ru Q. Xiong Q. Zhou M. Yue K. Wu Y. (2021). The role of chinese herbal therapy in methamphetamine abuse and its induced psychiatric symptoms. *Front. Pharmacol.* 12:679905. 10.3389/fphar.2021.679905 34040537 PMC8143530

[B8] Chen S. W. Min L. Li W. J. Kong W. X. Li J. F. Zhang Y. J. (2004). The effects of angelica essential oil in three murine tests of anxiety. *Pharmacol. Biochem. Behav.* 79 377–382. 10.1016/j.pbb.2004.08.017 15501315

[B9] Chen Y. Song R. Yang R. F. Wu N. Li J. (2014). A novel dopamine D3 receptor antagonist YQA14 inhibits methamphetamine self-administration and relapse to drug-seeking behaviour in rats. *Eur. J. Pharmacol.* 743 126–132. 25261038 10.1016/j.ejphar.2014.09.026

[B10] De La Garza R. II Mahoney J. J. III Culbertson C. Shoptaw S. Newton T. F. (2008). The acetylcholinesterase inhibitor rivastigmine does not alter total choices for methamphetamine, but may reduce positive subjective effects, in a laboratory model of intravenous self-administration in human volunteers. *Pharmacol. Biochem. Behav.* 89 200–208. 10.1016/j.pbb.2007.12.010 18207225 PMC4170947

[B11] Ferragud A. Velazquez-Sanchez C. Canales J. J. (2014). Modulation of methamphetamine’s locomotor stimulation and self-administration by JHW 007, an atypical dopamine reuptake blocker. *Eur. J. Pharmacol.* 731 73–79. 24675149 10.1016/j.ejphar.2014.03.015

[B12] Fu K. Lin H. Miyamoto Y. Wu C. Yang J. Uno K. (2016). Pseudoginsenoside-F11 inhibits methamphetamine-induced behaviors by regulating dopaminergic and GABAergic neurons in the nucleus accumbens. *Psychopharmacology* 233 831–840. 10.1007/s00213-015-4159-8 26621348

[B13] Glasner-Edwards S. Marinelli-Casey P. Hillhouse M. Ang A. Mooney L. J. Rawson R. (2009). Depression among methamphetamine users: association with outcomes from the methamphetamine treatment project at 3-year follow-up. *J. Nerv. Ment. Dis.* 197 225–231. 19363377 10.1097/NMD.0b013e31819db6fe PMC2749575

[B14] Gong X. Yue K. Ma B. Xing J. Gan Y. Wang D. (2016). Levo-tetrahydropalmatine, a natural, mixed dopamine receptor antagonist, inhibits methamphetamine self-administration and methamphetamine-induced reinstatement. *Pharmacol. Biochem. Behav.* 144 67–72. 10.1016/j.pbb.2016.01.010 26806555

[B15] Groman S. M. Lee B. Seu E. James A. S. Feiler K. Mandelkern M. A. (2012). Dysregulation of D(2)-mediated dopamine transmission in monkeys after chronic escalating methamphetamine exposure. *J. Neurosci.* 32 5843–5852. 10.1523/JNEUROSCI.0029-12.2012 22539846 PMC3353813

[B16] Guo W. Huang J. Wang N. Tan H. Y. Cheung F. Chen F. (2019). Integrating network pharmacology and pharmacological evaluation for deciphering the action mechanism of herbal formula zuojin pill in suppressing hepatocellular carcinoma. *Front. Pharmacol.* 10:1185. 10.3389/fphar.2019.01185 31649545 PMC6795061

[B17] Han J. Wan M. Ma Z. Hu C. Yi H. (2020). Prediction of targets of curculigoside a in osteoporosis and rheumatoid arthritis using network pharmacology and experimental verification. *Drug Design Dev. Therapy* 14 5235–5250. 10.2147/DDDT.S282112 33273808 PMC7705647

[B18] Harms R. Morsey B. Boyer C. W. Fox H. S. Sarvetnick N. (2012). Methamphetamine administration targets multiple immune subsets and induces phenotypic alterations suggestive of immunosuppression. *PLoS One* 7:e49897. 10.1371/journal.pone.0049897 23227154 PMC3515581

[B19] He D. Huang J. H. Zhang Z. Y. Du Q. Peng W. J. Yu R. (2019). A network pharmacology-based strategy for predicting active ingredients and potential targets of liuwei dihuang pill in treating type 2 diabetes mellitus. *Drug Design Dev. Therapy* 13 3989–4005. 10.2147/DDDT.S216644 31819371 PMC6890936

[B20] Ingram S. L. (2014). Pain: novel analgesics from traditional Chinese medicines. *Curr. Biol.* 24 R114–R116. 10.1016/j.cub.2013.12.030 24502784 PMC3980722

[B21] Jiaming D. Mei Z. Chaoying L. (2019). Improvement effects of jieduyihao on learning and memory impairment induced by methamphetamine in mice. *J. Jianghan Univ. (Nat. Sci. Ed.)* 47 66–72.

[B22] Krasnova I. N. Cadet J. L. (2009). Methamphetamine toxicity and messengers of death. *Brain Res. Rev.* 60 379–407. 10.1016/j.brainresrev.2009.03.002 19328213 PMC2731235

[B23] Kumar P. Kumar M. Wisdom K. S. Pathakota G. B. Nayak S. K. Reang D. (2020). Characterization, docking and molecular dynamics simulation of gonadotropin-inhibitory hormone receptor (gnihr2) in labeo catla. *Cell Physiol. Biochem.* 54 825–841. 10.33594/000000272 32871065

[B24] Lafuente J. V. Sharma A. Muresanu D. F. Ozkizilcik A. Tian Z. R. Patnaik R. (2018). Repeated forced swim exacerbates methamphetamine-induced neurotoxicity: neuroprotective effects of nanowired delivery of 5-ht3-receptor antagonist ondansetron. *Mol. Neurobiol.* 55 322–334. 10.1007/s12035-017-0744-7 28861718

[B25] Landa L. Jurajda M. Sulcova A. (2012). Altered dopamine D1 and D2 receptor mRNA expression in mesencephalon from mice exposed to repeated treatments with methamphetamine and cannabinoid CB1 agonist methanandamide. *Neuro Endocrinol. Lett.* 33 446–452. 22936253

[B26] Lee B. London E. D. Poldrack R. A. Farahi J. Nacca A. Monterosso J. R. (2009). Striatal dopamine d2/d3 receptor availability is reduced in methamphetamine dependence and is linked to impulsivity. *J. Neurosci.* 29 14734–14740. 10.1523/JNEUROSCI.3765-09.2009 19940168 PMC2822639

[B27] Limanaqi F. Gambardella S. Biagioni F. Busceti C. L. Fornai F. (2018). Epigenetic Effects Induced by Methamphetamine and Methamphetamine-Dependent Oxidative Stress. *Oxid. Med. Cell. Longev.* 2018:4982453. 10.1155/2018/4982453 30140365 PMC6081569

[B28] Meyer A. C. Horton D. B. Neugebauer N. M. Wooters T. E. Nickell J. R. Dwoskin L. P. (2011). Tetrabenazine inhibition of monoamine uptake and methamphetamine behavioral effects: locomotor activity, drug discrimination and self-administration. *Neuropharmacology* 61 849–856. 10.1016/j.neuropharm.2011.05.033 21669212 PMC3780357

[B29] Mi G. Gao Y. Yan H. Jin X. Ye E. Liu S. (2016). l-Scoulerine attenuates behavioural changes induced by methamphetamine in zebrafish and mice. *Behav. Brain Res.* 298(Pt A) 97–104. 10.1016/j.bbr.2015.09.039 26433144

[B30] Nakamura K. Sekine Y. Takei N. Iwata Y. Suzuki K. Anitha A. (2009). An association study of monoamine oxidase A (MAOA) gene polymorphism in methamphetamine psychosis. *Neurosci. Lett.* 455 120–123. 10.1016/j.neulet.2009.02.048 19368859

[B31] Nie L. Zhao Z. Wen X. Luo W. Ju T. Ren A. (2020). Gray-matter structure in long-term abstinent methamphetamine users. *BMC Psychiatry* 20:158. 10.1186/s12888-020-02567-3 32272912 PMC7146984

[B32] Nishio M. Kanda Y. Mizuno K. Watanabe Y. (2002). Methamphetamine increases the hippocampal alpha(2A)-adrenergic receptor and Galpha(o) in mice. *Neurosci. Lett.* 334 145–148. 10.1016/s0304-3940(02)01033-9 12453616

[B33] Nishio M. Kuroki Y. Watanabe Y. (2003). Role of hippocampal alpha(2A)-adrenergic receptor in methamphetamine-induced hyperlocomotion in the mouse. *Neurosci. Lett.* 341 156–160. 10.1016/s0304-3940(03)00171-x 12686389

[B34] Okita K. Morales A. M. Dean A. C. Johnson M. C. Lu V. Farahi J. (2018). Striatal dopamine D1-type receptor availability: no difference from control but association with cortical thickness in methamphetamine users. *Mol. Psychiatry* 23 1320–1327. 10.1038/mp.2017.172 28894300 PMC5847392

[B35] Park H. J. Kim H. Y. Yoon K. H. Kim K. S. Shim I. (2009). The effects of astragalus membranaceus on repeated restraint stress-induced biochemical and behavioral responses. *Korean J. Physiol. Pharmacol.* 13 315–319. 10.4196/kjpp.2009.13.4.315 19885016 PMC2766712

[B36] Paulus M. P. Stewart J. L. (2020). Neurobiology, clinical presentation, and treatment of methamphetamine use disorder: a review. *JAMA Psychiatry* 77 959–966. 10.1001/jamapsychiatry.2020.0246 32267484 PMC8098650

[B37] Peerzada H. Gandhi J. A. Guimaraes A. J. Nosanchuk J. D. Martinez L. R. (2013). Methamphetamine administration modifies leukocyte proliferation and cytokine production in murine tissues. *Immunobiology* 218 1063–1068. 10.1016/j.imbio.2013.02.001 23518444 PMC5589440

[B38] Peters K. Z. Oleson E. B. Cheer J. F. (2021). A brain on cannabinoids: the role of dopamine release in reward seeking and addiction. *Cold Spring Harbor Perspect. Med.* 11:a039305. 10.1101/cshperspect.a039305 31964646 PMC7778214

[B39] Prakash M. D. Tangalakis K. Antonipillai J. Stojanovska L. Nurgali K. Apostolopoulos V. (2017). Methamphetamine: effects on the brain, gut and immune system. *Pharmacol. Res.* 120 60–67. 10.1016/j.phrs.2017.03.009 28302577

[B40] Shin E. J. Dang D. K. Tran T. V. Tran H. Q. Jeong J. H. Nah S. Y. (2017). Current understanding of methamphetamine-associated dopaminergic neurodegeneration and psychotoxic behaviors. *Arch. Pharm. Res.* 40 403–428. 10.1007/s12272-017-0897-y 28243833

[B41] Su H. Sun T. Wang X. Du Y. Zhao N. Zhu J. (2020). Levo-tetrahydropalmatine attenuates methamphetamine reward behavior and the accompanying activation of ERK phosphorylation in mice. *Neurosci. Lett.* 714:134416. 10.1016/j.neulet.2019.134416 31398456

[B42] Sun L. Song R. Chen Y. Yang R. F. Wu N. Su R. B. (2016). A selective D3 receptor antagonist YQA14 attenuates methamphetamine-induced behavioral sensitization and conditioned place preference in mice. *Acta Pharmacol. Sin.* 37 157–165. 10.1038/aps.2015.96 26687935 PMC4753378

[B43] Tao W. Xu X. Wang X. Li B. Wang Y. Li Y. (2013). Network pharmacology-based prediction of the active ingredients and potential targets of Chinese herbal Radix Curcumae formula for application to cardiovascular disease. *J. Ethnopharmacol.* 145 1–10. 10.1016/j.jep.2012.09.051 23142198

[B44] Tian H. Liu Z. Pu Y. Bao Y. (2019). Immunomodulatory effects exerted by Poria Cocos polysaccharides *via* TLR4/TRAF6/NF-kappaB signaling *in vitro* and *in vivo*. *Biomed. Pharmacother.* 112:108709. 10.1016/j.biopha.2019.108709 30970514

[B45] Volkow N. D. Chang L. Wang G. J. Fowler J. S. Ding Y. S. Sedler M. (2001a). Low level of brain dopamine D2 receptors in methamphetamine abusers: association with metabolism in the orbitofrontal cortex. *Am. J. Psychiatry* 158 2015–2021. 10.1176/appi.ajp.158.12.2015 11729018

[B46] Volkow N. D. Chang L. Wang G. J. Fowler J. S. Franceschi D. Sedler M. (2001b). Loss of dopamine transporters in methamphetamine abusers recovers with protracted abstinence. *J. Neurosci.* 21 9414–9418. 10.1523/JNEUROSCI.21-23-09414.2001 11717374 PMC6763886

[B47] Volkow N. D. Chang L. Wang G. J. Fowler J. S. Leonido-Yee M. Franceschi D. (2001c). Association of dopamine transporter reduction with psychomotor impairment in methamphetamine abusers. *Am. J. Psychiatry* 158 377–382. 10.1176/appi.ajp.158.3.377 11229977

[B48] Volkow N. D. Wise R. A. Baler R. (2017). The dopamine motive system: implications for drug and food addiction. *Nat. Rev. Neurosci.* 18 741–752. 10.1038/nrn.2017.130 29142296

[B49] Wang G. J. Smith L. Volkow N. D. Telang F. Logan J. Tomasi D. (2012). Decreased dopamine activity predicts relapse in methamphetamine abusers. *Mol. Psychiatry* 17 918–925. 10.1038/mp.2011.86 21747399 PMC3261322

[B50] Xu W. Wang Y. Ma Z. Chiu Y. T. Huang P. Rasakham K. (2013). L-isocorypalmine reduces behavioral sensitization and rewarding effects of cocaine in mice by acting on dopamine receptors. *Drug Alcohol. Depend.* 133 693–703. 10.1016/j.drugalcdep.2013.08.021 24080315 PMC3954112

[B51] Yang C. Fu X. Hao W. Xiang X. Liu T. Yang B. Z. (2020). Gut dysbiosis associated with the rats’ responses in methamphetamine-induced conditioned place preference. *Addict. Biol.* 26:e12975. 10.1111/adb.12975 33094505

[B52] Yang G. Liu L. Zhang R. Li J. Leung C. K. Huang J. (2020). Cannabidiol attenuates methamphetamine-induced conditioned place preference *via* the Sigma1R/AKT/GSK-3beta/CREB signaling pathway in rats. *Toxicol. Res.* 9 202–211. 10.1093/toxres/tfaa021 32670551 PMC7329178

[B53] Yoo J. H. Cho J. H. Yu H. S. Lee K. W. Lee B. H. Jeong S. M. (2006). Involvement of 5-HT receptors in the development and expression of methamphetamine-induced behavioral sensitization: 5-HT receptor channel and binding study. *J. Neurochem.* 99 976–988. 10.1111/j.1471-4159.2006.04137.x 16942594

[B54] Yuan J. Lv R. Robert Brasic J. Han M. Liu X. Wang Y. (2014). Dopamine transporter dysfunction in Han Chinese people with chronic methamphetamine dependence after a short-term abstinence. *Psychiatry Res.* 221 92–96. 10.1016/j.pscychresns.2013.11.005 24314908

[B55] Yun J. (2014a). Limonene inhibits methamphetamine-induced locomotor activity *via* regulation of 5-HT neuronal function and dopamine release. *Phytomedicine* 21 883–887. 10.1016/j.phymed.2013.12.004 24462212

[B56] Yun J. (2014b). L-tetrahydropalmatine inhibits methamphetamine-induced locomotor activity *via* regulation of 5-HT neuronal activity and dopamine D3 receptor expression. *Phytomedicine* 21 1287–1291. 10.1016/j.phymed.2014.07.003 25172791

[B57] Zhang Z. He L. Huang S. Fan L. Li Y. Li P. (2018). Alteration of brain structure with long-term abstinence of methamphetamine by voxel-based morphometry. *Front. Psychiatry* 9:722. 10.3389/fpsyt.2018.00722 30618890 PMC6306455

[B58] Zhao N. Chen Y. Zhu J. Wang L. Cao G. Dang Y. (2014). Levo-tetrahydropalmatine attenuates the development and expression of methamphetamine-induced locomotor sensitization and the accompanying activation of ERK in the nucleus accumbens and caudate putamen in mice. *Neuroscience* 258 101–110. 10.1016/j.neuroscience.2013.11.025 24269936

[B59] Zhou Z. Y. Zhao W. R. Shi W. T. Xiao Y. Ma Z. L. Xue J. G. (2019). Endothelial-dependent and independent vascular relaxation effect of tetrahydropalmatine on rat aorta. *Front. Pharmacol.* 10:336. 10.3389/fphar.2019.00336 31057398 PMC6477965

[B60] Zulkipli N. N. Zakaria R. Long I. Abdullah S. F. Muhammad E. F. Wahab H. A. (2020). In silico analyses and cytotoxicity study of asiaticoside and asiatic acid from malaysian plant as potential mtor inhibitors. *Molecules* 25:3991. 10.3390/molecules25173991 32887218 PMC7504803

